# The Role of Bone Tacks in Sinus Floor Lift Surgery: A Single-Center Experience

**DOI:** 10.3390/jcm13154429

**Published:** 2024-07-29

**Authors:** Glauco Chisci, Luca Fredianelli, Maria Giulia Brunacci, Arjeta Hatia, Fabrizio Minichilli

**Affiliations:** 1 Centro Dentistico Chisci, Via Ricasoli 18, 58100 Grosseto, Italy; 2 Institute of Chemical and Physical Processes of National Research Council, Via G. Moruzzi 1, 56124 Pisa, Italy; 3 MOPI s.r.l., Via Cocchi 7, 56121 Pisa, Italy; mariagiuliabrunacci@mopilab.com; 4 Department of Dentistry, USL Toscana Sud-Est, 58100 Grosseto, Italy; arjeta.hatia@hotmail.it; 5 Clinical Physiology Institute, National Research Council, Via G. Moruzzi 1, 56124 Pisa, Italy; fabrizio.minichilli@cnr.it

**Keywords:** sinus floor lift, lateral sinus elevation, postoperative complications, dental implant, deproteinized bovine bone, bone tack, surgery, sinus floor elevation

## Abstract

**Background**: Maxillar atrophy is a prevalent condition associated with diminished bone volume, which precludes the conventional placement of dental implants. Sinus floor lift is a surgical procedure that aims to address this atrophy through the insertion of a graft within the sinus cavity. A multitude of techniques have been documented in the international literature for the management of the sinus bone window, though each approach has its own set of advantages and disadvantages. **Methods**: The present study is a retrospective analysis of traditional sinus floor lift surgery, comparing the outcomes of two surgical approaches: placement of a collagen membrane over the maxillary lateral bone window with or without bone tacks. The study enrolled a total of 48 consecutive patients. Twenty-four patients underwent sinus floor lift surgery, with the placement of a collagen membrane over the maxillary lateral bone window without bone tacks (control group). The remaining 24 patients underwent the same procedure, but with bone tacks (study group). All patients received an amoxicillin 875 mg + clavulanic acid 125 mg administration for six days and underwent Cone Beam Computed Tomography (CBCT) before the sinus floor lift surgery and six months later before the implant surgery. The parameters measured on the preoperative Cone Beam Computed Tomography (CBCT) scan included residual bone, the preoperative thickness of the lateral bone in the center, and the distance between the lateral wall and the medial wall of the sinus. Only the bone height increment was measured on the postoperative CBCT scan. Postoperative complications and the visual analogue scale (VAS) score were also recorded. A statistical analysis was performed, and the correlation between the parameters was evaluated using Pearson’s correlation coefficient. A comparison of the mean of the parameters between the treatment group and the control group was conducted using the *t*-test. **Results and Conclusions**: The study group was found to have superior outcomes in terms of bone height increment (*p* = 0.001) and VAS after 7 days (*p* = 0.11) compared to the control group. The novel application of bone tacks on the collagen membrane over the bone window following sinus elevation surgery was associated with superior outcomes in terms of bone height and reduced pain at seven days, as measured by the VAS, with no postoperative complications.

## 1. Introduction

At present, dental implants represent a standard and routinary procedure to restore edentulism. Once the teeth have been lost, dental implants in the maxillary or mandibular bone can be placed, allowing for the subsequent placement of the implant-supported prosthesis. Many aspects require attention in order to determine an implant treatment plan: systemic health, periodontitis, and patients taking medications. However, in cases of bone atrophy, the bone could be sufficient for dental implants, and preparatory procedures are required to have the correct amount of bone for the implants [1]. Furthermore, the study of bone morphology and comprehension of the boundaries of the implant surgery requires careful study of preoperative radiographs. Common radiographs in implant surgery for the study of bone morphology are panoramic radiograph and tridimensional Cone Beam Computed Tomography (CBCT). With regard to the maxillary bone, the presence of maxillary sinus often influences the placement of dental implants for the posterior teeth; the maxillary sinus is a cavern upon the alveolar bone and may be related to sinus pathology, as sinusitis and pseudocyst. Space upon the alveolar bone represents a limit when choosing the implant fixture; for this reason, sinus floor lift surgery was introduced many years ago, and it has become sufficiently reliable as a routine procedure for the treatment of atrophies of maxillary posterior areas [2,3,4]. The elevation of the sinus floor with the use of bone graft may increase the bone height when placing dental implants. In this case, implant surgery may be performed in the same surgical intervention or may be performed some months after the sinus lift elevation.

The regenerative procedures in oral surgery have been largely investigated in the last 30 years. The progress of these techniques focused on the evolution of graft materials, amount of bone gain, complications after sinus floor lift, and lateral or transalveolar sinus elevation [5,6,7,8,9,10]. The nature of bone graft combined with sinus floor lift has attracted considerable attention in the scientific community. Ferreira et al. [11] reported satisfactory results with inorganic bovine bone and collagen membrane in 406 sinus floor lifts. For many years, the autologous bone has represented a good choice for sinus floor lift surgery. However, many authors have underlined satisfactory results with deproteinized bovine bone in sinus floor lift surgery [7,9,12,13,14]. On the other hand, with regard to the end of the intervention after the bone graft, some authors report good results, with the bone window repositioning [15,16]. This concept of repositioning the cropped bone over the window aims to have a natural seal over the sinus, while, currently, the placement of a resorbable collagen membrane or nothing over the lateral maxillary bone window is common [17]. The placement of a seal over the lateral maxillary bone window at the end of the surgery, before the sutures, has the objective of preventing postoperative complications as small or large oroantral communications and reducing possible infection of the sinus graft from oral bacteria [18].

The present study evaluates the differences between sinus floor lift surgery performed with the placement of collagen membrane over the maxillary lateral bone window with versus without bone tacks. The aim is to identify possible differences in the efficacy of the use of bone tacks over the collagen membrane in preventing postoperative complications after sinus floor lift surgery and evaluate possible advantages and greater increase for bone augmentation.

## 2. Materials and Methods

The present retrospective study is based on data gathered from patients of the oral surgery service in an Italian dental center. In this regard, the standard procedure at the end of sinus floor lift surgery deployed in the study was changed from a placement of resorbable collagen membrane over the maxillary lateral bone window to a placement of resorbable collagen membrane fixed with two bone tacks over the maxillary lateral bone window. These procedures were compared by means of well-defined primary and secondary outcome measures. The groups were similar in biomaterials used and surgical techniques applied, with a difference emerging in terms of bone tacks’ placement. Patients who met the defined inclusion criteria and had received the placement of resorbable collagen membrane over the maxillary lateral bone window without bone tacks (control group) were matched to a placement of resorbable collagen membrane over the maxillary lateral bone window fixed with two bone tacks (study group).

The study involved 48 patients that received lateral sinus floor lift and delayed implant surgery between 2020 and 2023. Patients presented maxillary atrophy and partial edentulism as an indication of treatment and agreed to sinus floor lift surgery and delayed dental implant and prosthodontics. Patients were asked to sign a written informed consent form, in which treatment planning was discussed and a benefit/risk ratio was explicated, where the patients agreed to the processing of personal data and images for publishing purposes. The observational retrospective design did not require the approval of an ethics committee, as per Italian legislation on clinical investigations at the time of the study. Nevertheless, the investigation was carried out following the rules of the Declaration of Helsinki of 1975, revised in 2013, and performed according to the principles of the ICH Good Clinical Practice.

### 2.1. Inclusion/Exclusion Criteria

Eligible patients (inclusion criteria) were selected among those older than 18 years old, with partial edentulism, systemically healthy, with a diagnosis of maxillary atrophy and a treatment plan provided for sinus floor lift and delayed implant surgery. All of the patients were provided with a preoperative documentation of preoperative CBCT and a postoperative CBCT six months after sinus floor lift before the implant surgery.

The following exclusion criteria were considered during the present protocol: (i) requiring anticoagulation therapy; (ii) had systemic diseases that could interfere with oral tissue healing process/bleeding; (iii) were using bisphosphonates; (iv) were pregnant; (v) had mental/physical disabilities; (vi) underwent radiation treatment to the head or neck region; (vii) infection of the maxillary sinus or presence of pseudocyst or previous sinus surgery; (viii) periodontitis; (ix) smoking more than 5 cigarettes per day.

### 2.2. Sinus Floor Lift Surgery

All subjects underwent a session of oral prophylaxis hygiene, including instructions on correct oral hygiene measures and scaling; surgical treatment was not scheduled until the patient could demonstrate an adequate standard of supragingival plaque control. All patients received an amoxicillin 875 mg + clavulanic acid 125 mg administration 2 times per day for six days. Prior to surgery, patients were asked to rinse with a 0.2% chlorhexidine solution for one minute. All surgical procedures were performed by the same clinician (GC). Under local anesthesia, a full-thickness mucoperiosteal flap was elevated. The flap was made with an incision with a trapezoidal design from the mesial tooth to the edentulous ridge. Crestal and releasing incisions were beveled so that an increased connective surface would be available for sutures and for collagen membrane stabilization.

**Figure 1:**
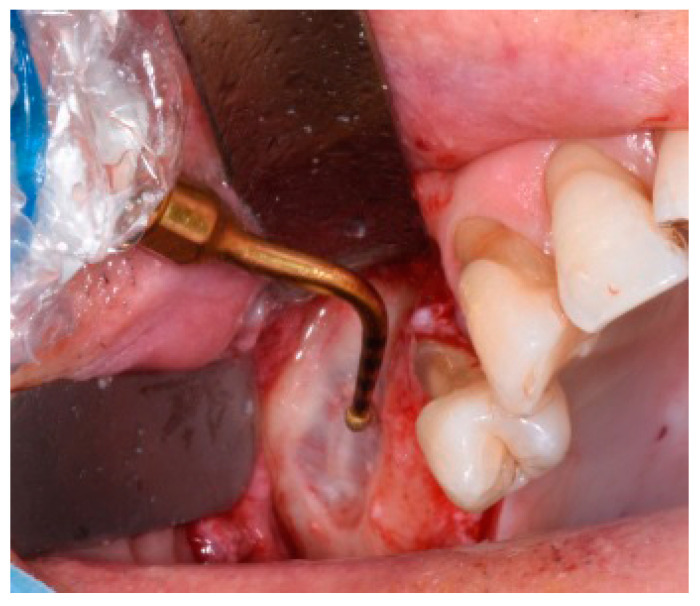
Intraoperative view during a sinus floor lift with piezoelectric device (Mectron Piezosurgery System, Genova, Italy).

**Figure 2:**
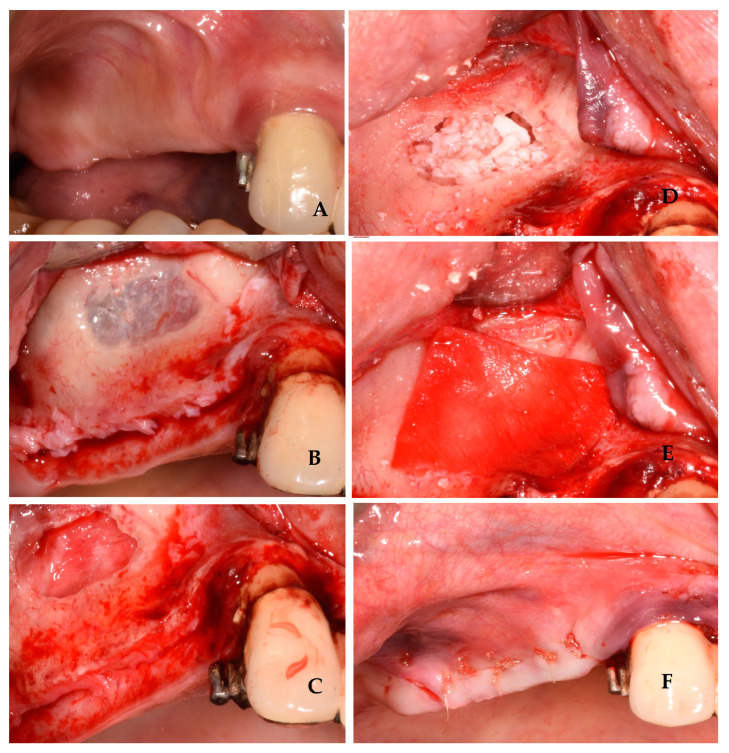
Sinus floor lift elevation surgery in the control group: preoperative view (**A**); intraoperative view with periosteal elevation and bone window performed (**B**); intraoperative view with sinusal Schneiderian membrane elevated (**C**); intraoperative view with bone graft filling inside the sinus (**D**); intraoperative view with bone window covered with a resorbable collagen membrane 13 × 25 mm (Bio-Gide Geistlich Pharma) (**E**); postoperative view with sutures (**F**).

**Figure 3:**
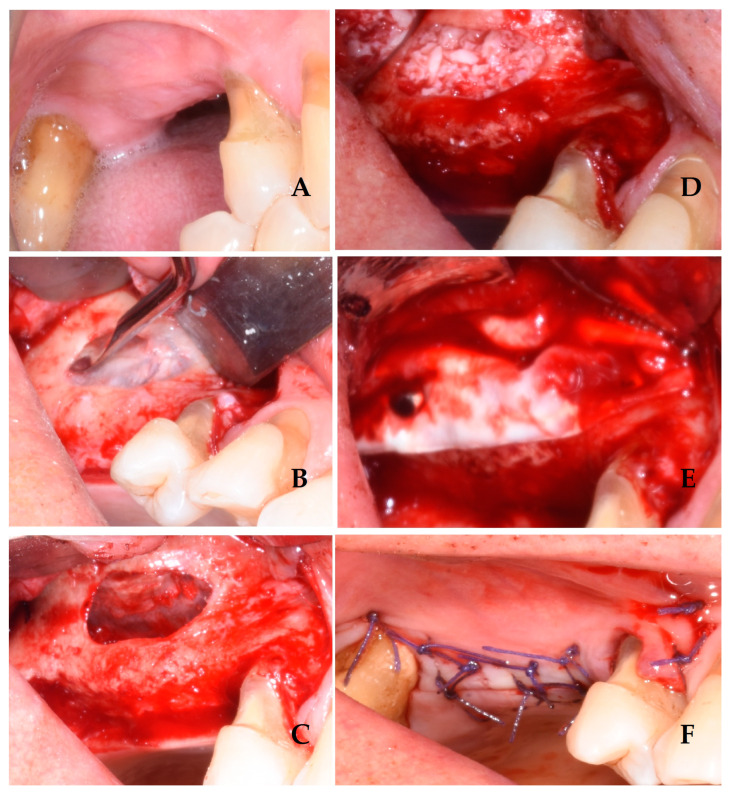
Sinus floor lift elevation surgery in the study group: preoperative view (**A**); intraoperative view with periosteal elevation and bone window performed during sinus membrane elevation (**B**); intraoperative view with sinusal Schneiderian membrane elevated (**C**); intraoperative view with bone graft filling inside the sinus (**D**); intraoperative view with bone window covered with a resorbable collagen membrane 13 × 25 mm (Bio-Gide Geistlich Pharma) and two bone tacks placed (Kalos, Nike, Orbetello, Italy) (**E**); postoperative view with sutures (**F**).

**Figure 4:**
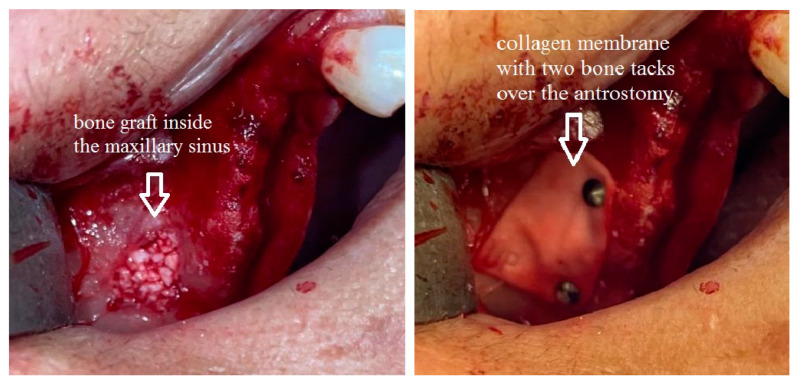
Detail of the sinus filling and the bone tacks.

Once the flap was raised, a bone window was opened, using the Mectron Piezosurgery System (Genova, Italy), to gain access to the maxillary sinus, as reported in Figure 1. The bony wall was reduced using an osteotomy tip until the Schneiderian membrane became evident in the fully shaved area, and bone window dimensions were approximated. The sinus membrane was lifted starting from the inferior border of the osteotomy site and completely and carefully dissected in the posterior, lateral, median, and mesial walls of the sinus. All surgical procedures were performed with great accuracy to avoid damage and perforation of the membrane. Once the sinus was elevated, the space was filled with deproteinized bovine bone (1 gr, particles 1–2 mm, Bio-oss, Geistlich Pharma Italy, Thiene, Italy) with manual filling, and the bony window was covered on the buccal surface with a resorbable collagen membrane 13 × 25 mm (Bio-Gide Geistlich Pharma Italy, Thiene, Italy), as reported in Figure 2. At this point, exclusively in the study group, two bone tacks were placed on the mesial and on the distal central surface of the membrane (Kalos, Nike, Orbetello, Italy), as reported in Figure 3 and Figure 4. Mattress sutures were placed on the crestal incision, and single stitches were placed in the release incisions (vicryl, ethicon, Johnson & Johnson, New York, NY, USA).

### 2.3. Post-Surgical and Follow-Up Protocols

The sutures were removed after 14 days. All patients received 2 gr of amoxicillin before starting the surgical procedure, and then this treatment was continued for 5 days (1 gr amoxicillin 2 times per day). Chlorhexidine 0.2% mouthwash was prescribed twice daily for the following 21 days. Dentures or provisionals were not permitted.

Patients were recalled at 7 days, 14 days, 30 days, and 180 days after sinus floor lift surgery. An oral hygiene assessment was performed every 6 months during the follow-up period. At the 6-month visit, a CBCT was performed, and implant surgery was scheduled. At the implant surgery interventions, the two bone tacks were gently removed.

### 2.4. Complications

A primary objective of this study was to determine if the study group could be inferior in terms of postoperative complication rate compared to the control group; for this purpose, events of dehiscence, sinusitis, and oroantral communications after sinus floor lift surgery were recorded both in the control and study groups and classified as minor or major complications: minor complications were classified as requiring medical treatment and no interfere with successive implant surgery; major complications were classified as requiring surgical treatment and planning a second sinus lift surgery. Intraoperative membrane perforations were recorded and treated with collagen membrane application.

### 2.5. Radiographic Evaluation

**Figure 5:**
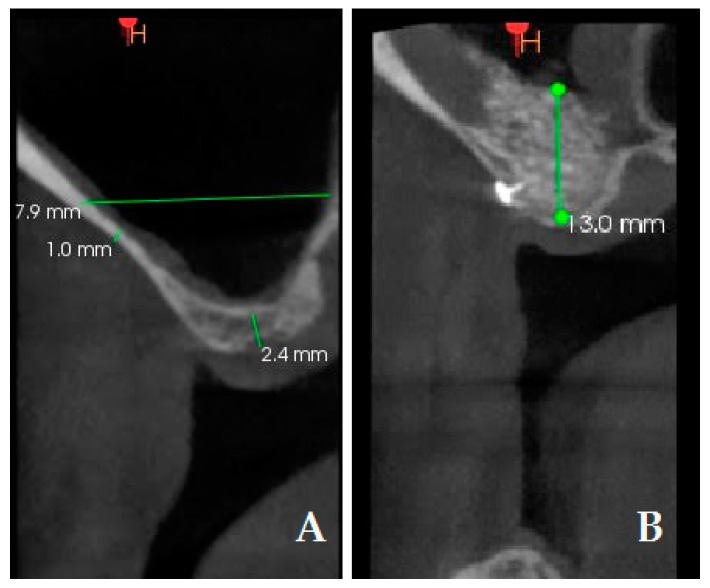
Cone Beam Computed Tomography (CBCT) measurements: preoperative residual bone (2.4 mm); preoperative thickness of lateral bone in the center (1 mm) (**A**); postoperative bone height (13 mm). A bone tack is observable in the postoperative CBCT (**B**).

Another primary objective of this study was to measure the height of the augmented bone between the test group and control group. Measurements were taken on the preoperative CBCT scans and 6-month postoperative CBCT scans (Figure 5). The following radiographic measurements were recorded on the preoperative CBCT in millimeters: (1) preoperative residual bone (PRB); (2) preoperative thickness of lateral bone in the center. Bone height increment (BHI) was recorded on the postoperative CBCT in mm and it was calculated as follows: 6-month bone height value—baseline residual height value.

### 2.6. Time for Surgical Procedure

A secondary outcome of this study was to determine whether there were differences in surgical intervention duration between the two tested procedures. A clinician, not involved in the surgical procedure, recorded all time-related outcomes. After administering local anesthesia, time was measured for each surgical procedure as follows: total time for sinus surgery (minutes); intraoperative partial time for bone window opening (seconds); intraoperative partial time for sinus elevation (seconds).

### 2.7. Visual Analog Scale (VAS)

Patient opinion was assessed using the VAS. All patients were asked to complete a questionnaire concerning their discomfort after the surgical intervention. All patients reported an overall judgment of the surgical treatment in terms of pain and swelling at 7 days (VAS7) and 14 days (VAS14). The VAS consisted of a 10 cm long line representing the spectrum of evaluation from 0 (no discomfort) to 100 (very important discomfort).

### 2.8. Statistical Analysis

After checking the normality of the data, the correlation between the parameters was evaluated with Pearson’s correlation coefficient. The comparison of the mean of the parameters between the treatment group and the control one was carried out through the unpaired *t*-test. The statistically significant results were defined as those with a *p*-value < 0.05. In agreement with recent studies to move beyond the concept of statistical significance [19], both statistically significant results and others for which only signals of association exist were reported in the Results section. The statistical analysis was performed by STATA 15.

## 3. Results

All patients presented at the follow-up visits, and all patients received the scheduled CBCTs and dental implants. All sinus floor lifts were successful, with the exception of two cases of minor complications that received medical treatment 1 month and 2 months, respectively, after sinus floor lift; both cases were referred to the control group. No cases of intraoperative complications, as membrane perforation, resulted in either the study or control groups. At the statistical analysis, a significant direct correlation was reported between VAS7 and VAS14 (r = 0.55, *p* = 0.011); a significant direct correlation was found between total time for sinus surgery and VAS7 (r = 0.37, *p* = 0.009). A significant direct correlation was found between intraoperative partial time for bone window and total time for sinus surgery (r = 0.38, *p* = 0.0257). No correlation was found between PRB and BHI. With regard to the comparison between the study group and control group, BHI resulted in being statistically higher in the study group compared to the control group (*p* = 0.001); no statistically significant differences resulted among the total time for sinus surgery between the study group and control group (*p* = 0.111); the mean VAS7 resulted in being lower with a statistically significant difference in the study group compared to the control group (*p* = 0.011); the mean VAS14 resulted in being lower in the study group compared to the control group but with no statistically significant difference (*p* = 0.65). Detailed data are reported in Table 1.

## 4. Discussion

Infection in oral and maxillofacial surgery after operations and antibiotic administration represents a great matter of interest for physicians [20]. With regard to sinus elevation surgery, Schlund et al. recently reported 217 sinus graft infections in 3319 patients, suggesting that antibiotics play a role in sinus lift elevation surgery [21]. Valentini and Artzi [22] reported similar considerations, suggesting the use of minimally invasive procedures for osteotomy of the maxillary lateral wall. The use of minimally invasive techniques is a modern approach in sinus elevation in oral and maxillofacial surgery. For this reason, much effort has been put into in vivo and in vitro research to introduce the use of a piezoelectric device for osteotomy, reporting better results in terms of a reduction in complications compared to classic osteotomy with a drill, with a slightly longer use of time [23,24]. Recently, Baldini et al. [25,26] introduced the use of minimally invasive trapezoidal flap and reduced bone window for sinus elevation surgery, reporting comparable results in terms of bone gain; the evolution of the sinus elevation technique leads to comparable results with lesser complications. With regard to suture and postoperative infection in sinus elevation surgery, few articles report data: Scarano et al. [27,28] reported interesting results on the study of bacterial adhesion to sutures. However, the kind of suture may affect microbial retention over the suture and possibly influence postoperative infection.

The present research focused on the collagen membrane seal after sinus elevation and before the suture; the results of the present research underline superior results in the study group compared to the control group in terms of bone height and VAS at 7 days. Furthermore, the only two complications that we reported were in the control group. With regard to the decision making on the bone window, at the beginning, in the international literature, some authors reported nothing or ipsilateral bone window as sinus seal [16,17]. Tawil et al. [15] reported many advantages with window repositioning in sinus elevation. The authors supported the idea that the need to spare the bone window limits the osteotomy technique and that some kind of fixation of the window is required and is strongly limited in cases of thin bone thickness. In all the cases reported, we used collagen membrane over the sinus window. Sim et al. [29] in rabbit models reported superior results in terms of bone height after sinus elevation in the groups with collagen membrane over the window compared to nothing over the window. On the other hand, Perini et al. [30], in their rabbit model study, reported comparable results with or without collagen membrane over the window, with increased bone quality near the bone walls and reduced bone quality near the window. In 2020, Imai et al. [31], in their interesting randomized study (collagen membrane vs. no collagen membrane), reported better results in the collagen membrane group, although with no statistically significant difference. In our study, bone height resulted in being statistically higher in the study group (collagen membrane with bone tacks) compared to the control group (collagen membrane without the bone tacks). As we support the idea that the single collagen membrane, that may move itself in the first hours after the operation over the periosteum plane, may not influence the rigidity of the blood clot and graft, our preliminary results suggest a role of the bone tacks to reduce this membrane mobility. The bone tacks act as an anchor to immobilize the collagen membrane and let the bone graft inside the sinus recover correctly. The use of bone tacks does not enlarge the invasiveness of this operation; we used two bone tacks, one on the mesial and one on the distal side of the collagen membrane on the bone window, in order to reduce the movements of the seal. These symmetric tacks are inserted in a thick bone, while in the upper side of the bone window, the bone commonly appears to be thinner. On the basis of our literature review, few articles report the use of bone tacks in sinus elevation surgery [32]. This research article appears to be the first preliminary study on the possible benefits of bone tacks on the collagen membrane on the bone window after sinus elevation surgery. The main limitations of this research are the retrospective modality and the reduced number of patients and the discrepancy between the study group and control group. Further, we advocate for a prospective randomized clinical trial to fulfill and confirm the results of this preliminary study. Lastly, the topic of this manuscript is indeed an important one, and we highlight its unique contribution to the existing body of literature.

## 5. Conclusions

In the present study, we reported our experience of improvement in the sinus lift surgical technique with regard to the use of bone tacks on the collagen membrane on the bone window after sinus elevation. We operated on 34 consecutive patients with preoperative and postoperative CBCT scans to evaluate the modifications of the studied variables and clinical symptoms of the patients. On the basis of our study, the study group with the use of bone tacks was associated with better results in terms of bone height and reduced pain at 7 days measured with VAS and no postoperative complications. The preliminary results of the present research suggest the use of bone tack in sinus elevation surgery in order to reduce postoperative symptoms and reduce infections. In order to obtain better results in terms of bone height increment, the use of bone tack could be helpful. The strengths of the present article are the routinary use of CBCT before and after the sinus elevation surgery and the standardization of the measures, as well as the innovative use of bone tacks in sinus elevation in order to obtain better results in terms of bone height. Future perspectives of this research could include the performance of a randomized study with the same parameters used here with more and matched patients.

## Figures and Tables

**Table 1:** Measured variables of sinus lift surgery.

Variables		Group		
		Control	Test	*p*
		n = 24	n = 24	
Preoperative residual bone height (mm)	Mean	2.45	2.95	0.171
	Std. Err.	0.21	0.30	
	Std. Dev	1.00	1.45	
Preoperative thickness of the lateral wall in the center (mm)	Mean	1.38	1.56	0.130
	Std. Err.	0.13	0.18	
	Std. Dev	0.61	0.87	
Bone height increment (mm)	Mean	9.41	12.64	0.001
	Std. Err.	0.31	0.42	
	Std. Dev	1.53	2.07	
Intraoperative partial time for bone	Mean	171.46	158.67	0.262
	Std. Err.	7.01	8.83	
	Std. Dev	34.33	43.27	
Intraoperative partial time for sinus opening (seconds)	Mean	207.17	215.08	0.312
	Std. Err.	3.13	7.08	
	Std. Dev	15.32	34.66	
Total time for sinus surgery (minutes)	Mean	50.31	49.06	0.436
	Std. Err.	0.97	1.27	
	Std. Dev	4.76	6.20	
VAS7	Mean	4.42	3.29	0.011
	Std. Err.	0.33	0.36	
	Std. Dev	1.64	1.76	
VAS14	Mean	1.04	0.96	0.655
	Std. Err.	0.21	0.23	
	Std. Dev	1.04	1.12	

## Data Availability

Data are contained within the article.
